# Choline metabolism and its implications in cancer

**DOI:** 10.3389/fonc.2023.1234887

**Published:** 2023-11-01

**Authors:** Nan Yao, Wenqiang Li, Guoshuai Xu, Ning Duan, Guoyong Yu, Jun Qu

**Affiliations:** ^1^ Department of General Surgery, Aerospace Center Hospital, Beijing, China; ^2^ Department of Nephrology, Beijing University of Chinese Medicine Affiliated Dongzhimen Hospital, Beijing, China

**Keywords:** choline, metabolism, cancer, diagnosis, treatment

## Abstract

Choline, a quintessential quaternary ammonium compound, plays a cardinal role in several pivotal biological mechanisms, chiefly in safeguarding cell membrane integrity, orchestrating methylation reactions, and synthesizing vital neurotransmitters. This systematic review meticulously dissects the complex interplay between choline metabolism and its profound implications in oncology. The exposition is stratified into three salient dimensions: Initially, we delve into the intricacies of choline metabolism, accentuating its indispensability in cellular physiology, the enzymatic labyrinth governing its flux, and the pivotal cellular import mechanisms. Subsequently, we elucidate the contemporary comprehension of choline metabolism in the cancer paradigm, traversing its influence from inception to the intricate metamorphosis during oncogenic progression, further compounded by dysregulated enzyme activities and aberrant signaling cascades. Conclusively, we illuminate the burgeoning potential of choline-centric metabolic imaging modalities, notably magnetic resonance spectroscopy (MRS) and positron emission tomography (PET), as avant-garde tools for cancer diagnostics and therapeutic trajectory monitoring. Synoptically, the nuanced perturbations in choline metabolism in neoplastic entities unfurl critical insights, potentially heralding paradigm shifts in diagnostic and therapeutic oncological stratagems. A deeper foray into this realm is anticipated to fortify our molecular understanding and refine intervention modalities in cancer theranostics.

## Introduction

Choline, a type of quaternary ammonium compound, is a vital nutrient involved in numerous biological functions, such as maintaining cell membrane integrity, facilitating methylation reactions, and aiding in the synthesis of neurotransmitters (1, 2). It is a key component of phosphatidylcholine (PtdCho), a primary element of cell membranes, and plays a role in the creation of acetylcholine, a neurotransmitter that is crucial for nerve function (3). Choline can either be derived from the food we consume or be synthesized within our bodies (4). The liver is central to choline metabolism, where it is converted into phosphocholine and subsequently into PtdCho (5). Other organs, such as the brain, also have the capacity to produce choline, albeit to a lesser degree. Beyond its role in cellular structure and function, choline also contributes to methylation reactions. It provides methyl groups in the creation of S-adenosylmethionine, a universal methyl donor involved in the methylation of DNA, proteins, and lipids (6). This process is vital for the regulation of gene expression and protein function (6). Choline metabolism is interconnected with other metabolic pathways, including those of methionine and folate (7). Methionine serves as a precursor of S-adenosylmethionine, while folate is involved in the regeneration of methionine from homocysteine (8). The interaction between these pathways highlights the complexity of choline metabolism and its significance in cellular function.

Recent research has shown that choline metabolism undergoes significant changes in cancer, resulting in a heightened demand for choline and its metabolites (9). The rapid growth of cancer cells necessitates an increased production of PtdCho for the formation of new cell membranes (5, 10). Furthermore, altered signaling pathways in cancer cells enhance the uptake and utilization of choline. These changes in choline metabolism have been linked to the onset of cancer, advancement of tumors, and resistance to treatment (11, 12). Moreover, the use of choline-based metabolic imaging has shown great potential as a tool for both detecting and monitoring several types of cancer (13, 14).

Choline metabolism shifts in cancer are not solely attributed to a heightened need for choline and its byproducts. These alterations also originate from modifications in the expression and functionality of enzymes involved in the metabolism of choline (8). In this systematic review, we aim to investigate the relationships between choline metabolism and its implications in cancer through the following three aspects: 1) choline metabolism; 2) current understanding of choline metabolism in cancer; and 3) use of choline-based metabolic imaging for cancer diagnosis and treatment monitoring.

## Choline metabolism: an overview

Choline metabolism plays a central role in numerous cell functions, including the creation of cell membranes, single-carbon metabolism, and cholinergic neurotransmission (15). The metabolic pathway of choline is regulated by a series of enzymes, each contributing to the overall balance of choline and its derivatives within the cell (16).The first step in choline metabolism is its uptake into the cell, a process facilitated by choline transporters (17, 18). These transporters are proteins that are embedded in the cell membrane and function to transport choline from the extracellular environment into the cell. This process is vital for maintaining the intracellular concentration of choline and ensuring that the cell has sufficient choline to meet its metabolic needs (17, 18). When choline enters the cell, it is phosphorylated by choline kinase alpha (CHKα), resulting in the production of PtdCho (19). This stage is considered the pace-setting step in the production of PtdCho, a primary component of the cell membrane. PtdCho is vital for preserving the stability of the cell membrane and contributes to cell signaling and lipid transportation (20).

Phosphocholine cytidylyltransferase (CCT) then facilitates the conversion of PtdCho and cytidine triphosphate into cytidine 5’-diphosphocholine (CDP-choline) (21, 22). CDP-choline, the most activated choline intermediate in the Kennedy pathway, is directly utilized by diacylglycerol cholinephosphotransferase 1 to generate PtdCho (22). CCT exists in both a dormant soluble state and an active lipid-attached state within the nuclear membrane. Its activity is regulated by fluctuations in the intracellular concentration of choline and its byproducts. Besides these enzymes, choline metabolism is also affected by other enzymes like phosphatidylethanolamine N-methyltransferase, which transforms phosphatidylethanolamine into PtdCho, thereby restoring cellular PtdCho levels (23). Other enzymes, including phospholipase A2, PtdCho-specific phospholipase D, and PtdCho-specific phospholipase C, participate in the breakdown of PtdCho, yielding free choline and other metabolites. These enzymes are vital in maintaining the equilibrium of choline and its metabolic products within the cell (23, 24).

## Choline metabolism in cancer: current understanding

### Choline metabolism in cancer initiation

Limited research has been conducted on the association between choline metabolism and cancer risk, with a focus primarily on dietary choline intake rather than plasma choline levels. In the available literature, plasma choline is mostly negatively correlated with new-onset cancer risk, including colorectal cancer (25, 26), and pancreatic cancer (27), while one study indicates an increased risk of CRC associated with plasma choline levels (28). However, these studies are primarily nested case-control studies with their own inherent limitations, such as selection bias, and small sample size.

### Choline metabolism in cancer progression

**Figure 1:**
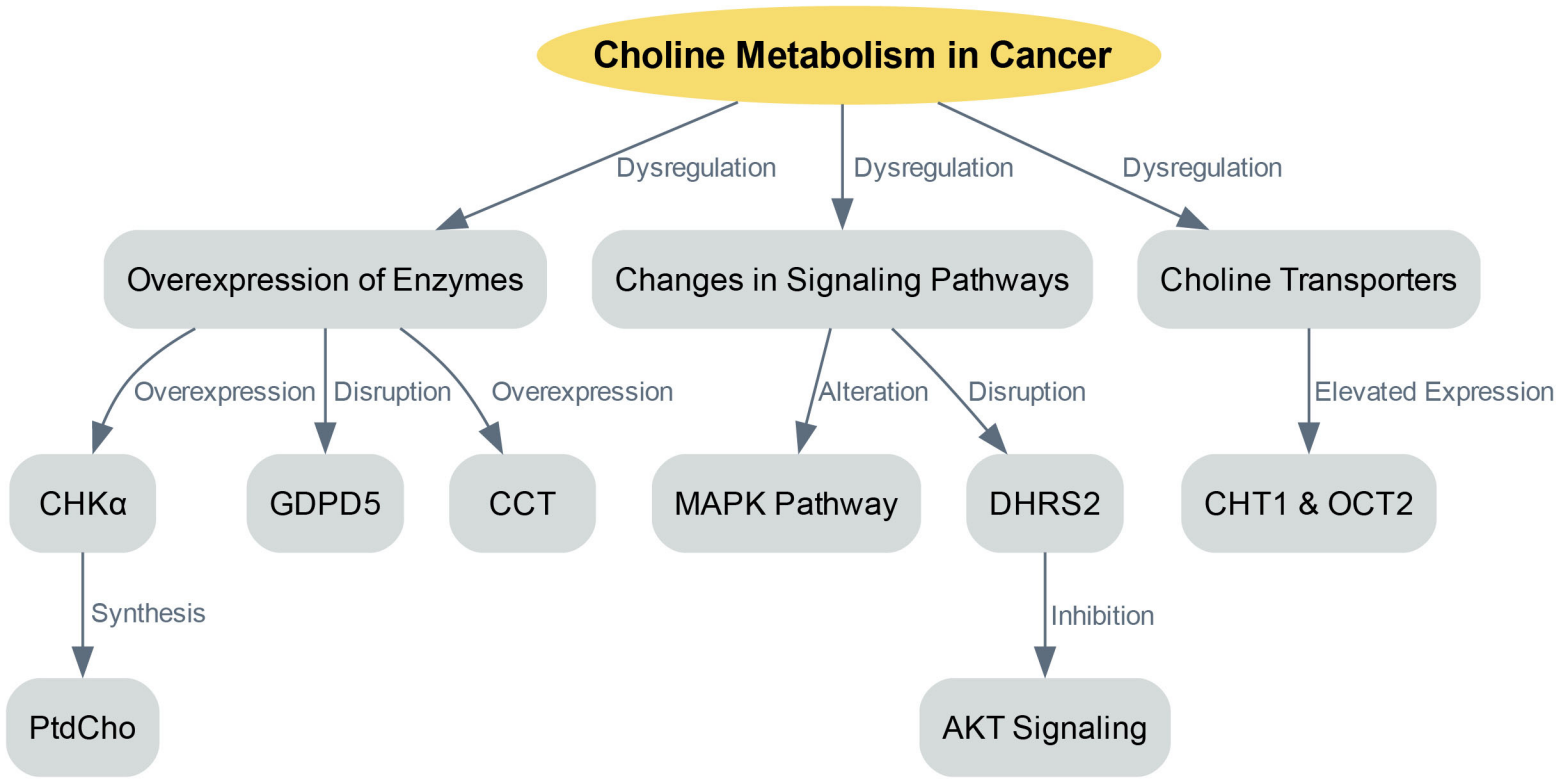
Choline metabolism in cancer progression.

The dysregulation of choline metabolism plays a significant role in the initiation of cancer. This complexity arises from several factors, including the overexpression of enzymes involved in choline metabolism, changes in signaling pathways that promote choline uptake and utilization, and variations in choline transporters (**Figure 1**).

One key factor in choline dysregulation is the overexpression of enzymes involved in choline metabolism, such as CHKα. CHKα is crucial as it is essential for the synthesis of PtdCho, a primary component of cellular membranes. Various cancers have shown overexpression of CHKα, leading to an increased production of PtdCho, which supports rapid cell proliferation (15). Besides CHKα, other enzymes involved in choline metabolism also exhibit disruptions in cancer. For example, glycerophosphodiester phosphodiesterase domain containing 5 (GDPD5) plays a role in regulating choline metabolism. Overexpression of GDPD5 has been linked to enhanced choline uptake and utilization, contributing to the dysregulation of choline metabolism in cancer (29). Furthermore, the onset of liver cancer has been associated with increased CCT activity and mRNA expression (21). Similarly, elevated CCT expression and activity have been observed in colon cancer, leading to higher PtdCho levels (22).

In cancer, choline metabolism becomes dysregulated not only due to enzyme overexpression but also because of changes in signaling pathways that enhance choline uptake and utilization (5, 30). The MAPK pathway, which is a central regulator of various cellular processes, is altered in cancer, leading to increased choline uptake and use. The activation of CHKα and elevated PtdCho levels were first observed in NIH3T3 fibroblasts stimulated with serum and transformed with either KRAS or HRAS. The activation of the MAPK pathway by oncogenic HRAS results in the regulation of CCT transcription (11). Another study integrated transcriptome and metabolome data and found that dehydrogenase/reductase member 2 (DHRS2) disrupts choline metabolism in ovarian cancer (31). Specifically, DHRS2 post-transcriptionally downregulates CHKα, inhibiting AKT signaling activation, which in turn leads to a reduced PtdCho/glycerophosphorylcholine ratio. This disruption in choline metabolism reprogramming is a primary factor behind DHRS2’s tumor-suppressive effect in ovarian cancer.

Choline transporters are another crucial component of choline metabolism in cancer (32). These transporters are in charge of absorbing free choline from the surroundings, a process that can be a pace-setting stage in the creation of PtdCho and within the Kennedy pathway (32). The human genome contains four sets of proteins capable of transporting choline: organic cation/carnitine transporters, choline transporter 1 (CHT1/SLC5A7), organic cation transporters (OCTs) and choline transporter-like proteins (10, 33). In order to secure the uptake of this essential nutrient, cancer cells typically express at least one category of choline transporters (34, 35). The expression levels of choline transporter genes and the speed of choline absorption in cancer cells are frequently much greater than in non-cancerous cells (35). For instance, breast cancer cell lines have exhibited elevated expression of CHT1 and OCT2 when compared to non-cancerous human mammary epithelial cells (33). The heightened requirement for choline in cancer cells implies their potential susceptibility to inhibition of choline transporters. Organic cation drugs, which probably use the same transporters as choline, could hinder choline absorption, thus decreasing the viability of cancer cells. For example, the choline analogue hemicholinium-3 and tetrahexylammonium chloride have been documented to hinder choline absorption and decrease cell growth in specific cancer cell lines (35).

### Therapeutic strategies targeting choline metabolism

Choline metabolism is emerging as a pivotal focus in cancer research, offering promising therapeutic avenues. The inhibition of oncogenic signaling pathways with specific anticancer drugs induces changes in choline-containing metabolite levels, underscoring its therapeutic potential (32). Delving deeper, aberrations in choline metabolism in cancers have been identified, paving the way for innovative treatments targeting this metabolic pathway (36). The role of choline phospholipid metabolism is accentuated when considering its molecular targets, which present opportunities for groundbreaking anticancer therapies (37). Furthermore, the potential of this metabolic pathway is magnified when enzymes, such as choline kinase, are targeted, heralding a new era in anticancer treatment strategies (38).

In conclusion, the disruption of choline metabolism in cancer is a complex process involving multiple factors. Gaining a comprehensive understanding of this process can yield valuable insights into the underlying biology of cancer and potentially pave the way for novel approaches in cancer diagnosis and treatment (35).

## Choline-based metabolic imaging for cancer diagnosis and treatment monitoring

Choline-based metabolic imaging has emerged as a promising tool for the detection and monitoring of cancer, offering potential applications in cancer diagnosis and treatment assessment (39). This approach leverages the alterations in choline metabolism that occur in cancerous cells, providing a unique avenue for non-invasive cancer detection and monitoring (35, 40). As mentioned previously, cancer cells often exhibit dysregulated choline metabolism, leading to an increased demand for choline and its metabolites. Metabolic imaging techniques based on choline, such as magnetic resonance spectroscopy (MRS) and positron emission tomography (PET) and fluorescence imaging, have been developed to exploit these metabolic alterations for cancer diagnosis and treatment monitoring (41–43). These techniques allow for non-invasive visualization and quantification of choline and its metabolites in tumors, providing valuable information about tumor biology and response to therapy (42). PET imaging using radio-tagged choline has proven to be successful in identifying a range of cancers, such as liver and prostate cancer (44, 45). This technique provides high-resolution images of choline uptake in tumors, allowing for accurate localization and staging of the disease. Moreover, choline PET imaging can be employed to track the reaction to treatment, as changes in choline uptake can reflect alterations in tumor metabolism and growth (46). Similarly, MRS can be used to measure the levels of choline and its metabolites in tumors (42). This technique provides a metabolic profile of the tumor, which can provide insights into tumor biology and response to therapy (42). For instance, an increase in the choline peak in MRS spectra has been linked to tumor advancement, while a decrease in the choline peak has been associated with response to therapy (47). Also, fluorescence imaging is a very convenient method to monitor CHKα *in vivo* (48). **Table 1** highlights the most relevant studies focused on choline-based metabolic imaging for cancer diagnosis and treatment monitoring.

**Table 1:** The relevant studies linking the choline-based metabolic imaging for cancer diagnosis and treatment monitoring.

Study	Design	Samples, n	Findings related to this topic
Glunde K, 2011 (39)	Review	NA	MRS techniques can be used in diagnosis, treatment identification, monitoring, and response assessment to enhance treatment outcomes. Combined with new molecular imaging probes and functional imaging capabilities, MRS is valuable for cancer research and drug discovery.
Iorio E, 2016 (40)	Review	NA	The changes in choline metabolism in cancer cells offer a distinctive path for non-invasive detection and monitoring of cancer.
Cheng M, 2016 (35)	Review	NA	Numerous studies have explored the molecular causes of altered choline metabolism, aiming to identify cancer treatment targets and refine MRS methods for clinical diagnosis and monitoring.
Pysz MA, 2010 (41)	Review	NA	Molecular imaging uses PET and SPECT to detect diseases early and personalize treatment, with ongoing research developing new targets, agents, and imaging strategies for clinical use in the future.
Brindle KM, 2011 (42)	Review	NA	Dynamic nuclear polarization improves cancer imaging and spectroscopy for 13C-labeled cell metabolites, but human imaging challenges need to be resolved before it can be applied to cancer patient management.
Arlauckas SP, 2014 (43)	Experimental study	NA	JAS239, a newly developed carbocyanine dye, can inhibit the activity of choline kinase alpha (ChoK) in breast cancer cells, leading to cell death, and it has the potential to be used as a companion diagnostic tool for noninvasive breast tumor staging and as a novel treatment for aggressive, therapy-resistant tumors.
Wetter A, 2017 (44)	Cross-sectional study	22	PET/MRI integration enables concurrent PET and MR spectroscopy, revealing a meaningful association between choline compounds and metabolism.
Kolthammer JA, 2011 (45)	Experimental study	NA	Both (11)C-choline and (18)F-fluoroethylcholine can be used for PET imaging of hepatocellular carcinoma (HCC), and fasting did not impact the accumulation of either tracer. These findings support additional research into the potential clinical applications of FEC for HCC imaging.
Phan LM, 2014 (46)	Review	NA	Altered choline uptake can indicate changes in tumor metabolism and proliferation.
Verma N, 2013 (47)	Review	NA	The elevation of the choline peak in magnetic resonance spectroscopy spectra is linked to tumor progression, whereas a reduction in the choline peak is connected to a positive response to therapy.
Arlauckas SP, 2017 (48)	Experimental study	NA	A ChoKα inhibitor called JAS239 could be used as a new method to monitor effective inhibitors of choline metabolism in breast cancer, which distinguishes tumors and delineates tumor margins in vivo.

MRS, magnetic resonance spectroscopy; PET, positron emission tomography; SPECT, single photon emission computed tomography; ChoK, choline kinase alpha; HCC, hepatocellular carcinoma; MRI, magnetic resonance imaging; NA, not applicable.

In conclusion, choline-based metabolic imaging holds promise as a valuable tool for detecting and monitoring cancer, offering potential applications in both cancer diagnosis and treatment evaluation. By leveraging the alterations in choline metabolism that occur in cancer, these techniques provide a unique avenue for non-invasive cancer detection and monitoring. However, further research is needed to optimize these techniques and to fully elucidate the implications of choline metabolism in cancer.

## Conclusions

The intricate interplay between choline metabolism and cancer opens up new avenues for advancements in cancer detection and therapeutic interventions. The disruption of choline metabolism in cancer cells has been linked to the onset of cancer, advancement of tumors, and resistance to therapy, implying that choline metabolism could serve as a viable target for both cancer detection and therapeutic interventions. The potential of choline-based metabolic imaging for cancer diagnosis and treatment monitoring is currently being explored, with promising results. However, additional research is required to completely clarify the mechanisms behind the changes in choline metabolism in cancer and to develop effective choline-based diagnostic and therapeutic strategies. The complexity of choline metabolism and its importance in various biological processes underscore the need for a multifaceted approach to cancer research. By integrating insights from different fields, including biochemistry, molecular biology, and imaging science, we can gain a more comprehensive understanding of the role of choline metabolism in cancer and develop more effective strategies for cancer diagnosis and treatment.

## Author contributions

All authors contributed to the article and approved the submitted version. NY: Writing-Original draft preparation. WL: Writing-Reviewing and Editing. GX and ND: Writing-Reviewing and Editing. GY and JQ: Supervision.

## References

[1] Kansakar U Trimarco V Mone P Varzideh F Lombardi A Santulli G. Choline supplements: An update. Front Endocrinol (2023) 14:1148166. doi: 10.3389/fendo.2023.1148166 PMC10025538 36950691

[2] Obeid R Derbyshire E Schön C. Association between maternal choline, fetal brain development, and child neurocognition: systematic review and meta-analysis of human studies. Adv Nutr (Bethesda Md) (2022) 13(6):2445–57. doi: 10.1093/advances/nmac082 PMC9776654 36041182

[3] Zeisel SH. Gene response elements, genetic polymorphisms and epigenetics influence the human dietary requirement for choline. IUBMB Life (2007) 59(6):380–7. doi: 10.1080/15216540701468954 PMC2430110 17613168

[4] Obeid R. The metabolic burden of methyl donor deficiency with focus on the betaine homocysteine methyltransferase pathway. Nutrients (2013) 5(9):3481–95. doi: 10.3390/nu5093481 PMC3798916 24022817

[5] Ducker GS Rabinowitz JD. One-carbon metabolism in health and disease. Cell Metab (2017) 25(1):27–42. doi: 10.1016/j.cmet.2016.08.009 27641100 PMC5353360

[6] Walker AK Jacobs RL Watts JL Rottiers V Jiang K Finnegan DM. A conserved SREBP-1/phosphatidylcholine feedback circuit regulates lipogenesis in metazoans. Cell (2011) 147(4):840–52. doi: 10.1016/j.cell.2011.09.045 PMC3384509 22035958

[7] Ramos-Lopez O Milagro FI Allayee H Chmurzynska A Choi MS Curi R. Guide for current nutrigenetic, nutrigenomic, and nutriepigenetic approaches for precision nutrition involving the prevention and management of chronic diseases associated with obesity. J nutrigen nutrigenom (2017) 10(1-2):43–62. doi: 10.1159/000477729 28689206

[8] Kaushik AK DeBerardinis RJ. Applications of metabolomics to study cancer metabolism. Biochim Biophys Acta Rev Cancer (2018) 1870(1):2–14. doi: 10.1016/j.bbcan.2018.04.009 29702206 PMC6193562

[9] Van Puyvelde H Dimou N Katsikari A Indave Ruiz BI Godderis L Huybrechts I. The association between dietary intakes of methionine, choline and betaine and breast cancer risk: A systematic review and meta-analysis. Cancer Epidemiol (2023) 83:102322. doi: 10.1016/j.canep.2023.102322 36701983

[10] Inazu M. Choline transporter-like proteins CTLs/SLC44 family as a novel molecular target for cancer therapy. Biopharma Drug dispos (2014) 35(8):431–49. doi: 10.1002/bdd.1892 24532461

[11] Wang X Zheng Z Caviglia JM Corey KE Herfel TM Cai B. Hepatocyte TAZ/WWTR1 promotes inflammation and fibrosis in nonalcoholic steatohepatitis. Cell Metab (2016) 24(6):848–62. doi: 10.1016/j.cmet.2016.09.016 PMC5226184 28068223

[12] Kroemer G Pouyssegur J. Tumor cell metabolism: cancer's Achilles' heel. Cancer Cell (2008) 13(6):472–82. doi: 10.1016/j.ccr.2008.05.005 18538731

[13] Kurhanewicz J Vigneron DB Males RG Swanson MG Yu KK Hricak H. The prostate: MR imaging and spectroscopy. Present future. Radiol Clinics North America (2000) 38(1):115–138, viii-ix. doi: 10.1016/S0033-8389(05)70152-4 10664669

[14] Meisamy S Bolan PJ Baker EH Bliss RL Gulbahce E Everson LI. Neoadjuvant chemotherapy of locally advanced breast cancer: predicting response with in *vivo* (1)H MR spectroscopy–a pilot study at 4 T. Radiology (2004) 233(2):424–31. doi: 10.1148/radiol.2332031285 15516615

[15] Glunde K Penet MF Jiang L Jacobs MA Bhujwalla ZM. Choline metabolism-based molecular diagnosis of cancer: an update. Expert Rev Mol diagn (2015) 15(6):735–47. doi: 10.1586/14737159.2015.1039515 PMC4871254 25921026

[16] Sanchez-Lopez E Zhong Z Stubelius A Sweeney SR Booshehri LM Antonucci L. Choline uptake and metabolism modulate macrophage IL-1β and IL-18 production. Cell Metab (2019) 29(6):1350–1362.e1357. doi: 10.1016/j.cmet.2019.03.011 30982734 PMC6675591

[17] Bagnoli M Granata A Nicoletti R Krishnamachary B Bhujwalla ZM Canese R. Choline metabolism alteration: A focus on ovarian cancer. Front Oncol (2016) 6:153. doi: 10.3389/fonc.2016.00153 27446799 PMC4916225

[18] Snider SA Margison KD Ghorbani P LeBlond ND O'Dwyer C Nunes JRC. Choline transport links macrophage phospholipid metabolism and inflammation. J Biol Chem (2018) 293(29):11600–11. doi: 10.1074/jbc.RA118.003180 PMC6065184 29880645

[19] Arlauckas SP Popov AV Delikatny EJ. Choline kinase alpha-Putting the ChoK-hold on tumor metabolism. Prog Lipid Res (2016) 63:28–40. doi: 10.1016/j.plipres.2016.03.005 27073147 PMC5360181

[20] Pessi G Choi JY Reynolds JM Voelker DR Mamoun CB. *In vivo* evidence for the specificity of Plasmodium falciparum phosphoethanolamine methyltransferase and its coupling to the Kennedy pathway. J Biol Chem (2005) 280(13):12461–6. doi: 10.1074/jbc.M414626200 15664981

[21] Yang WS SriRamaratnam R Welsch ME Shimada K Skouta R Viswanathan VS. Regulation of ferroptotic cancer cell death by GPX4. Cell (2014) 156(1-2):317–31. doi: 10.1016/j.cell.2013.12.010 PMC4076414 24439385

[22] Dixon SJ Lemberg KM Lamprecht MR Skouta R Zaitsev EM Gleason CE. Ferroptosis: an iron-dependent form of nonapoptotic cell death. Cell (2012) 149(5):1060–72. doi: 10.1016/j.cell.2012.03.042 PMC3367386 22632970

[23] Xiong J Bian J Wang L Zhou JY Wang Y Zhao Y. Dysregulated choline metabolism in T-cell lymphoma: role of choline kinase-α and therapeutic targeting. Blood Cancer J (2015) 5(3):287. doi: 10.1038/bcj.2015.10 25768400 PMC4382653

[24] Popov AV Mawn TM Kim S Zheng G Delikatny EJ. Design and synthesis of phospholipase C and A2-activatable near-infrared fluorescent smart probes. Bioconjugate Chem (2010) 21(10):1724–7. doi: 10.1021/bc100271v PMC2958237 20882956

[25] Bae S Ulrich CM Neuhouser ML Malysheva O Bailey LB Xiao L. Plasma choline metabolites and colorectal cancer risk in the Women's Health Initiative Observational Study. Cancer Res (2014) 74(24):7442–52. doi: 10.1158/0008-5472.CAN-14-1835 PMC4268282 25336191

[26] Guertin KA Li XS Graubard BI Albanes D Weinstein SJ Goedert JJ. Serum trimethylamine N-oxide, carnitine, choline, and betaine in relation to colorectal cancer risk in the alpha tocopherol, beta carotene cancer prevention study. Cancer epidemiol Biomarkers Prev Publ Am Assoc Cancer Res cosponsored by Am Soc Prev Oncol (2017) 26(6):945–52. doi: 10.1158/1055-9965.EPI-16-0948 PMC5608021 28077427

[27] Huang JY Luu HN Butler LM Midttun Ø Ulvik A Wang R. A prospective evaluation of serum methionine-related metabolites in relation to pancreatic cancer risk in two prospective cohort studies. Int J Cancer (2020) 147(7):1917–27. doi: 10.1002/ijc.32994 PMC11537248 32222976

[28] Nitter M Norgård B de Vogel S Eussen SJ Meyer K Ulvik A. Plasma methionine, choline, betaine, and dimethylglycine in relation to colorectal cancer risk in the European Prospective Investigation into Cancer and Nutrition (EPIC). Ann Oncol (2014) 25(8):1609–15. doi: 10.1093/annonc/mdu185 24827130

[29] Cao MD Döpkens M Krishnamachary B Vesuna F Gadiya MM Lønning PE. Glycerophosphodiester phosphodiesterase domain containing 5 (GDPD5) expression correlates with Malignant choline phospholipid metabolite profiles in human breast cancer. NMR biomed (2012) 25(9):1033–42. doi: 10.1002/nbm.2766 PMC4126590 22279038

[30] Turski ML Thiele DJ. New roles for copper metabolism in cell proliferation, signaling, and disease. J Biol Chem (2009) 284(2):717–21. doi: 10.1074/jbc.R800055200 PMC2613604 18757361

[31] Li Z Tan Y Li X Quan J Bode AM Cao Y. DHRS2 inhibits cell growth and metastasis in ovarian cancer by downregulation of CHKα to disrupt choline metabolism. Cell Death Dis (2022) 13(10):845. doi: 10.1038/s41419-022-04540-2 36192391 PMC9530226

[32] Glunde K Bhujwalla ZM Ronen SM. Choline metabolism in Malignant transformation. Nat Rev Cancer (2011) 11(12):835–48. doi: 10.1038/nrc3162 PMC4337883 22089420

[33] Inazu M Yamada T Kubota N Yamanaka T. Functional expression of choline transporter-like protein 1 (CTL1) in small cell lung carcinoma cells: a target molecule for lung cancer therapy. Pharmacol Res (2013) 76:119–31. doi: 10.1016/j.phrs.2013.07.011 23948665

[34] Iorio E Ricci A Bagnoli M Pisanu ME Castellano G Di Vito M. Activation of phosphatidylcholine cycle enzymes in human epithelial ovarian cancer cells. Cancer Res (2010) 70(5):2126–35. doi: 10.1158/0008-5472.CAN-09-3833 PMC2831129 20179205

[35] Cheng M Bhujwalla ZM Glunde K. Targeting phospholipid metabolism in cancer. Front Oncol (2016) 6:266. doi: 10.3389/fonc.2016.00266 28083512 PMC5187387

[36] Glunde K Jacobs MA Bhujwalla ZM. Choline metabolism in cancer: implications for diagnosis and therapy. Expert Rev Mol diagn (2006) 6(6):821–9. doi: 10.1586/14737159.6.6.821 17140369

[37] Glunde K Serkova NJ. Therapeutic targets and biomarkers identified in cancer choline phospholipid metabolism. Pharmacogenomics (2006) 7(7):1109–23. doi: 10.2217/14622416.7.7.1109 17054420

[38] Glunde K Ackerstaff E Mori N Jacobs MA Bhujwalla ZM. Choline phospholipid metabolism in cancer: consequences for molecular pharmaceutical interventions. Mol pharma (2006) 3(5):496–506. doi: 10.1021/mp060067e 17009848

[39] Glunde K Bhujwalla ZM. Metabolic tumor imaging using magnetic resonance spectroscopy. Semin Oncol (2011) 38(1):26–41. doi: 10.1053/j.seminoncol.2010.11.001 21362514 PMC3275885

[40] Iorio E Caramujo MJ Cecchetti S Spadaro F Carpinelli G Canese R. Key players in choline metabolic reprograming in triple-negative breast cancer. Front Oncol (2016) 6:205. doi: 10.3389/fonc.2016.00205 27747192 PMC5043614

[41] Pysz MA Gambhir SS Willmann JK. Molecular imaging: current status and emerging strategies. Clin Radiol (2010) 65(7):500–16. doi: 10.1016/j.crad.2010.03.011 PMC3150531 20541650

[42] Brindle KM Bohndiek SE Gallagher FA Kettunen MI. Tumor imaging using hyperpolarized 13C magnetic resonance spectroscopy. Magnet resonance Med (2011) 66(2):505–19. doi: 10.1002/mrm.22999 21661043

[43] Arlauckas SP Popov AV Delikatny EJ. Direct inhibition of choline kinase by a near-infrared fluorescent carbocyanine. Mol Cancer Ther (2014) 13(9):2149–58. doi: 10.1158/1535-7163.MCT-14-0085 PMC4209917 25028471

[44] Wetter A Grüneisen J Fliessbach K Lütje S Schaarschmidt B Umutlu L. Choline-based imaging of prostate cancer with combined [(18)F] fluorocholine PET and (1)H MR spectroscopy by means of integrated PET/MRI. Clin Imaging (2017) 42:198–202. doi: 10.1016/j.clinimag.2016.12.008 28110202

[45] Kolthammer JA Corn DJ Tenley N Wu C Tian H Wang Y. PET imaging of hepatocellular carcinoma with 18F-fluoroethylcholine and 11C-choline. Eur J Nucl Med Mol Imaging (2011) 38(7):1248–56. doi: 10.1007/s00259-011-1743-y 21344223

[46] Phan LM Yeung SC Lee MH. Cancer metabolic reprogramming: importance, main features, and potentials for precise targeted anti-cancer therapies. Cancer Biol Med (2014) 11(1):1–19. doi: 10.7497/j.issn.2095-3941.2014.01.001 24738035 PMC3969803

[47] Verma N Cowperthwaite MC Burnett MG Markey MK. Differentiating tumor recurrence from treatment necrosis: a review of neuro-oncologic imaging strategies. Neuro-oncology (2013) 15(5):515–34. doi: 10.1093/neuonc/nos307 PMC3635510 23325863

[48] Arlauckas SP Kumar M Popov AV Poptani H Delikatny EJ. Near infrared fluorescent imaging of choline kinase alpha expression and inhibition in breast tumors. Oncotarget (2017) 8(10):16518–30. doi: 10.18632/oncotarget.14965 PMC5369982 28157707

